# PROX1-mediated epigenetic silencing of *SIRT3* contributes to proliferation and glucose metabolism in colorectal cancer

**DOI:** 10.7150/ijbs.73530

**Published:** 2023-01-01

**Authors:** Lu Gan, Qingguo Li, Wei Nie, Yi Zhang, Hesheng Jiang, Cong Tan, Long Zhang, Jieyun Zhang, Qian Li, Pengcong Hou, Yitao Yuan, Xun Sun, Dongmei Liu, Weiqi Sheng, Tianshu Liu, Midie Xu, Weijian Guo

**Affiliations:** 1 Department of Gastrointestinal Medical Oncology, Fudan University Shanghai Cancer Center, Shanghai, 200032, China.; 2 Department of Oncology, Shanghai Medical College, Fudan University, Shanghai, 200032, China.; 3 Department of Medical Oncology, Zhongshan Hospital, Fudan University, Shanghai, 200032, China.; 4 Department of Colorectal Surgery, Fudan University Shanghai Cancer Center, Shanghai, 200032, China.; 5 Department of Pulmonary Medicine, Shanghai Chest Hospital, School of Medicine, Shanghai Jiao Tong University, Shanghai, 200032, China.; 6 Department of Gastroenterology & Clinical Nutrition, The 452nd Hospital of PLA, Chengdu 610000, Sichuan, China.; 7 Department of Surgery, United Health Services Southern California Medical Education Consortium, Temecula Valley Hospital, Temecula, CA 92592, USA.; 8 Department of Pathology, Fudan University Shanghai Cancer Center, Shanghai, 200032, China.; 9 Institute of Pathology, Fudan University, Shanghai, 200032, China.

**Keywords:** PROX1, *SIRT3*, EZH2, colorectal cancer, aerobic glycolysis, prognosis

## Abstract

Prospero-related homeobox 1 (PROX1) is a homeobox transcription factor known to promote malignant transformation and stemness in human colorectal cancer (CRC). However, the biological function of PROX1 in metabolic rearrangement in CRC remains unclear. Here, we aimed to uncover the relationship between the expression profile and role of PROX1 and CRC cell glucose metabolism and to elucidate the underlying molecular mechanism. PROX1 expression was significantly upregulated in human CRC tissues and positively associated with the maximum standardized uptake value (SUVmax), a measure of tissue 18-fluoro-2-deoxy-D-glucose uptake and an indicator of glycolysis and tumor cell activity, in patients with CRC. Knockdown of PROX1 suppressed CRC cell proliferation and glucose metabolism *in vitro* and *in vivo*. Mechanistically, through a physical interaction, PROX1 recruited EZH2 to the SIRT3 promoter and inhibited *SIRT3* promoter activity. Moreover, PROX1 or EZH2 knockdown decreased cell glycolysis by targeting *SIRT3*. Clinically, high PROX1 expression combined with low *SIRT3* expression predicted poor prognosis in patients with CRC. Thus, our study suggests that the PROX1-EZH2 complex positively regulates cell proliferation and glucose metabolism by engaging *SIRT3* in CRC, which may serve as a promising therapeutic strategy for CRC.

## Introduction

Colorectal cancer (CRC) is one of the highest-incidence cancers and the leading cause of cancer-related death worldwide 1; specifically, CRC ranks third in incidence and mortality in both men and women worldwide, and the relative 5-year survival rate for CRC is 65% 1, 2. Despite recent advances in CRC screening techniques and therapy, the overall survival (OS) of patients diagnosed with stage IV disease remains poor, with a 5-year survival rate of 11% 3. The main reason for the failure of colorectal cancer treatment is metastasis, which leads to prognosis and short survival time. The most common treatment for metastatic colorectal cancer (mCRC) is systemic chemotherapy and molecular targeted drugs. Nevertheless, for patients with asymptomatic primary lesion and synchronous unresectable metastases, induction chemotherapy followed by primary tumor resection can't bring survival benefits 4; and currently applied standard second-line chemotherapy regimen, such as FOLFIRI (folinic acid, fluorouracil, and irinotecan), has been proved increases not efficacy but toxicity in patients with mCRC 5. In recent years, there has been considerable progress in the research and development of checkpoint inhibitors for mCRC with microsatellite-high (MSI-H) status and drugs targeting BRAF-mutant CRC. However, there is still a need to identify key genes participating in CRC growth and metastasis to support the development of combination therapy strategies.

Aerobic glycolysis provides cancerous cells with building blocks for macromolecule synthesis and helps generate an acidic extracellular microenvironment, leading to extracellular matrix destruction that favors metastasis 6. Furthermore, glycolytic genes have been reported to play direct regulatory roles in cancer cell proliferation and metastasis; for example, pyruvate kinase M2 (PKM2) sustains cancer cell proliferation 7 and contributes to gefitinib resistance in colon cancer cells 8.

Prospero-related homeobox 1 (PROX1), an evolutionarily conserved member of the homeobox transcription factor family, regulates cell differentiation and development during embryogenesis 9. Importantly, PROX1 has been found to induce the neoplastic transformation of normal epithelial cells in CRC 10. In addition, PROX1 is overexpressed and associated with several tumor proliferation-associated genes, and its high expression confers worse prognosis of CRC 11-13. PROX1 has been reported to interact with a panel of genes implicated in glucose metabolism, including β-catenin 14 and DBC1 15, in CRC 11, 16, and PROX1 has been implicated in the metabolic adaptation of metastatic colon cancer cells 17. Specifically, PROX1 positively regulates the protein stability of HIF1α, a master regulator of glycolysis 18, and regulates gluconeogenesis and cholesterol metabolism 19. Collectively, these discoveries suggest the role of PROX1 in metabolic reprogramming in cancer. However, it is unclear how PROX1 and its downstream effectors affect cancer cell glucose metabolism and how this ultimately affects CRC carcinogenesis and progression.

These findings raise questions regarding the downstream effects of functional PROX1 on cancer cell glucose metabolism that result in CRC carcinogenesis and progression. To study whether PROX1 is related to tumor glucose metabolism, we analyzed the association between PROX1 expression in human tumors and glycolysis, measured as the maximum standardized uptake value (SUVmax), and patient prognosis. We also determined the impact of PROX1 on proliferation and glucose metabolism in functional *in vitro* and *in vivo* mouse experiments and explored the downstream targets and interacting partners by gene expression profiling analysis and proteomic screening.

## Materials and Methods

### Human tissue specimens

A total of 217 CRC samples, including cancer tissue and paired adjacent normal colorectal epithelial samples, were enrolled for immunohistochemistry (IHC) analysis. All patients' formalin-fixed paraffin-embedded (FFPE) tissues were obtained from the Department of Pathology of Fudan University Shanghai Cancer Center (FUSCC) between 2011 and 2013. The tumor grades were defined in accordance with the criteria outlined by the World Health Organization (WHO) Classification of Tumors of the Digestive System, 2010 edition. The study complied with the regulations of the Ministry of Health of China, the WHO Research Ethics Review Committee international guidelines for research involving human subjects and the Declaration of Helsinki on the Ethical Principles for Medical Research Involving Human Subjects.

Proteomic (four main phosphoprotein sites of PRXO1: S79, Y80, S179 and S511) and mRNA data from the Clinical Proteomic Tumor Analysis Consortium (CPTAC) and The Cancer Genome Atlas (*TCGA*) CRC cohorts were downloaded via the UALCAN online database (http://ualcan.path.uab.edu/analysis-prot.html) 20.

### Tissue preparation and immunostaining

Immunostaining of mouse tumor and tissue microarray (TMA) sections was conducted as previously described 21. Antibodies against PROX1 (ab199359, 1:500 dilution), EZH2 (ab191080, 1:250 dilution) and *SIRT3* (ab189860, 1:50 dilution) for IHC were obtained from Abcam. PBS with matched IgG was used as a negative control. Experiments were performed as described previously, and each sample was scored by using an immunoreactive score (IRS) method that combines the values of immunoreaction intensity and the percentage of tumor cell staining as described previously 22. Protein expression was defined based on the IRS as low (≤1+) or high (>2+ to ≤3+).

### Chromatin immunoprecipitation (ChIP) assays

ChIP assays were performed as previously described 23. PCR primer sequences are listed in **Supplemental Table S6**. Ten percent of the combined supernatants were kept as the input for the second ChIP.

### Whole-body 18F-fluorodeoxyglucose (FDG) positron emission tomography/computed tomography (PET/CT)

Images of PET/CT were acquired from 71 patients with CRC on a Siemens Biograph 16HR PET/CT scanner with a transaxial intrinsic spatial resolution of 4.1 mm. The quantification of metabolic activity was performed using the standardized uptake value (SUV) normalized to body weight, and the SUVmax for each lesion was calculated.

### RNA-sequencing data analysis

Total RNA (1 μg) was isolated from SW480 cells and treated with VAHTS mRNA Capture Beads (Vazyme, Nanjing, China) to enrich polyA+ RNA before constructing the RNA libraries. RNA library preparation was performed by using a VAHTS mRNA-sequencing v2 Library Prep Kit for Illumina (Vazyme, Nanjing, China). Paired-end sequencing was performed with an Illumina HiSeq 3000 at RiboBio Co., Ltd. (Guangzhou, China). For computational analysis of RNA-sequencing data, sequencing reads were aligned using the spliced read aligner HISAT2, which was supplied with the Ensemble human genome assembly (Genome Reference Consortium GRCh38) as the reference genome. Gene expression levels were calculated by fragments per kilobase of transcript per million mapped reads (FPKM). Gene set enrichment analysis (GSEA), a bioinformatic method used to assess whether sets of genes are significantly different, was performed. The method was used to compute the similarity between a query gene set compared to the gene sets available in the GSEA database and derived from published studies. The Java GSEA Desktop Application (http://www.broadinstitute.org/gsea/index.jsp) was used with the hallmark gene set collections.

### Luciferase assays

Cells were transfected with pGL3-based constructs containing the *SIRT3* promoter plus the Renilla luciferase plasmid (pRL-TK). The cells were harvested after 48 h for firefly/Renilla luciferase assays using the Dual-Luciferase Reporter Assay System (Promega). Luciferase activities were normalized to the cotransfected pRL-TK plasmid (mean ± SD).

Other methods used in this study were described in previous publications and are listed in the Supplementary Information 21, 23, 24.

### Reproducibility

Each experiment was performed in triplicate, and the data are presented as the mean ± SD. The results for the sphere formation, cell invasion, animal experimental, western blot, ChIP, PET/CT and immunohistochemistry analyses are representative of three independent experiments.

### Statistical analysis

All statistical analyses were performed using SPSS 24.0 (IBM, SPSS, Chicago, IL, USA) and GraphPad Prism version 7.0 (GraphPad Software, San Diego, CA, USA). Statistical tests for comparing data between groups included the χ^2^ test, Student's two-tailed t test and one-way ANOVA, as appropriate. The disease-specific survival (DFS) rate was calculated from the date of surgery to the date of progression (local and/or distal tumor recurrence) or to the date of death. The OS rate was defined as the length of time between diagnosis and death or last follow-up. The Kaplan-Meier method with the log-rank test was used to calculate the DFS and OS curves. Univariate and multivariate analyses were fit using a Cox proportional hazards regression model. A threshold of P < 0.05 was defined as statistically significant.

## Results

### PROX1 expression is positively correlated with glucose metabolism, tumor progression and outcomes in CRC patients

**Figure 1:**
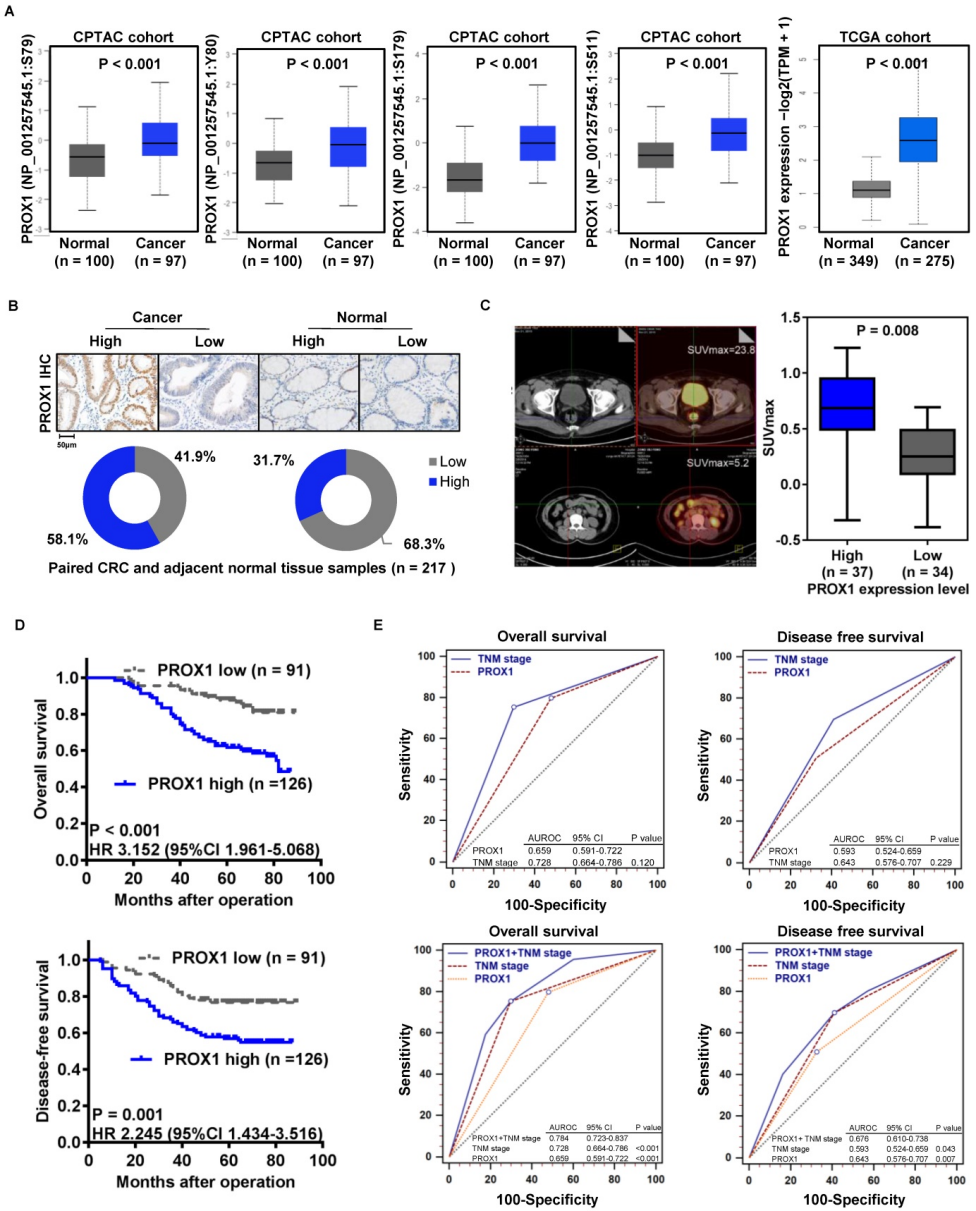
** Increased PROX1 expression is positively correlated with the ^18^F-FDG PET/CT SUVmax and predicts poor prognosis of CRC. A.** Box plots comparing PROX1 protein expression levels (four main phosphoprotein sites) and mRNA expression levels in the CPTAC and *TCGA* cohorts downloaded from the UALCAN online database. The PROX1 proteomic expression profile is shown as the Z value, and the PROX1 mRNA expression profile is shown as log2(TPM + 1) and was analyzed using one-way *ANOVA*. **B.** Representative images (upper panel, 10× and 200×) and distribution analysis (lower panel) of PROX1 immunohistochemical staining in CRC and adjacent normal colorectal samples with high and low levels. **C.** Representative 18F-FDG PET/CT imaging of CRC patients (magnification scale bar, 20 µm) and analysis of the SUVmax in PROX1^low^ (n = 34) and PROX1^high^ groups (n = 37). **D.** Kaplan-Meier analysis of the correlation of PROX1 expression with OS and DFS. Log-rank tests were used to determine statistical significance. **E.** Receiver operating characteristic (ROC) curves for DFS and OS. P values show the area under the ROC (AUROC) of the PROX1 signature versus the AUROC of TNM stage (upper panel) and the AUROC of the combined PROX1 expression and TNM stage model versus AUROCs of TNM stage alone or PROX1 expression alone (lower panel).

To confirm that PROX1 protein expression is associated with CRC carcinogenesis, we compared the four main phosphoprotein site expression levels of PROX1 protein in CRC and normal colorectal tissue samples using proteomic and genomic data from the CPTAC CRC and *TCGA* cohorts via the UALCAN online database (http://ualcan.path.uab.edu/analysis-prot.html) 20. PROX1 expression was significantly higher in CRC tissue than in normal colorectal epithelial samples in the CPTAC and *TCGA* CRC cohorts (**Figure 1A**). To confirm the clinical significance of PROX1 in CRC, we analyzed PROX1 protein expression in 217 paired tumor tissues and adjacent normal colorectal epithelial tissues (from the FUSCC cohort) by IHC. By calculating the IRS, we found that PROX1 immunostaining was high in 58.1% (n = 126) and low in 41.9% (n = 91) of CRC samples, whereas in normal colorectal epithelial samples, PROX1 immunostaining was low in 68.3% (n = 148) and high in 31.7% (n = 69) (**Figure 1B**). Among the 217 patients with CRC, 71 underwent preoperative PET/CT imaging. Thirty-seven samples were defined as patients with high PROX1 expression based on the mean expression value of PROX1 mRNA, and the other 34 samples were defined as patients with low PROX1 expression. We further investigated whether PROX1 protein levels correlated with the SUVmax, which indicates the metabolic activity of tumor lesions, from these 71 CRC patients. The SUVmax was significantly higher in patients with high PROX1 immunostaining (n = 37) than in those with low PROX1 immunostaining (n = 34, P = 0.008; **Figure 1C**). These data suggest that PROX1 is significantly upregulated in CRC and positively correlated with tumor glucose metabolism.

Moreover, analysis of the correlation of PROX1 levels with clinicopathological data for patients with CRC showed that high PROX1 expression was positively associated with tumor depth of invasion (P = 0.031), lymphatic metastasis (P = 0.001), TNM stage (P = 0.002) and Ki67 immunostaining (P = 0.049; **Table 1**). Given the clinical association of PROX1 expression with tumor progression in our cohort, we aimed to determine whether PROX1 expression is also a prognostic factor in CRC. Thus, we evaluated the correlation between PROX1 immunostaining and CRC patient prognosis by Kaplan-Meier analysis with the log-rank test. Patients with high PROX1 expression had significantly worse OS (P < 0.001) and DFS (P = 0.001, **Figure 1D**) than patients with low PROX1 expression. Furthermore, we compared the prognostic value of PROX1 and TNM stage for DFS and OS using receiver operating characteristic (ROC) curves and found no differences between these factors (**Figure 1E, upper panels**). Interestingly, the prognostic value of PROX1 expression plus TNM stage was better than that of either factor alone (**Figure 1E, lower panels**), suggesting that improved predictive accuracy for CRC should be obtained by combining PROX1 expression and TNM stage assessments.

Univariate Cox proportional hazards analysis showed that the PROX1 and Ki67 IHC scores; tumor differentiation (histologic grade) and size; vascular invasion; lymphatic metastasis; and TNM stage were prognostic factors for OS and DFS in CRC patients (**Tables 2-3**). Multivariate Cox proportional hazards analysis revealed that histologic grade (HR 1.447, P = 0.022), lymphatic metastasis (HR 3.906, P = 0.000) and PROX1 IHC score (HR 2.275, P = 0.008) were independent prognostic factors for OS in CRC patients (**Table 2**). In addition, histologic grade (HR 1.489, P = 0.008), lymphatic metastasis (HR 3.985, P = 0.000) and ki67 IHC score (HR 2.789, P = 0.003) were independent prognostic factors for DFS in CRC patients (**Table 3**).

### Knockdown of PROX1 inhibits CRC cell proliferation and glycolysis

**Figure 2:**
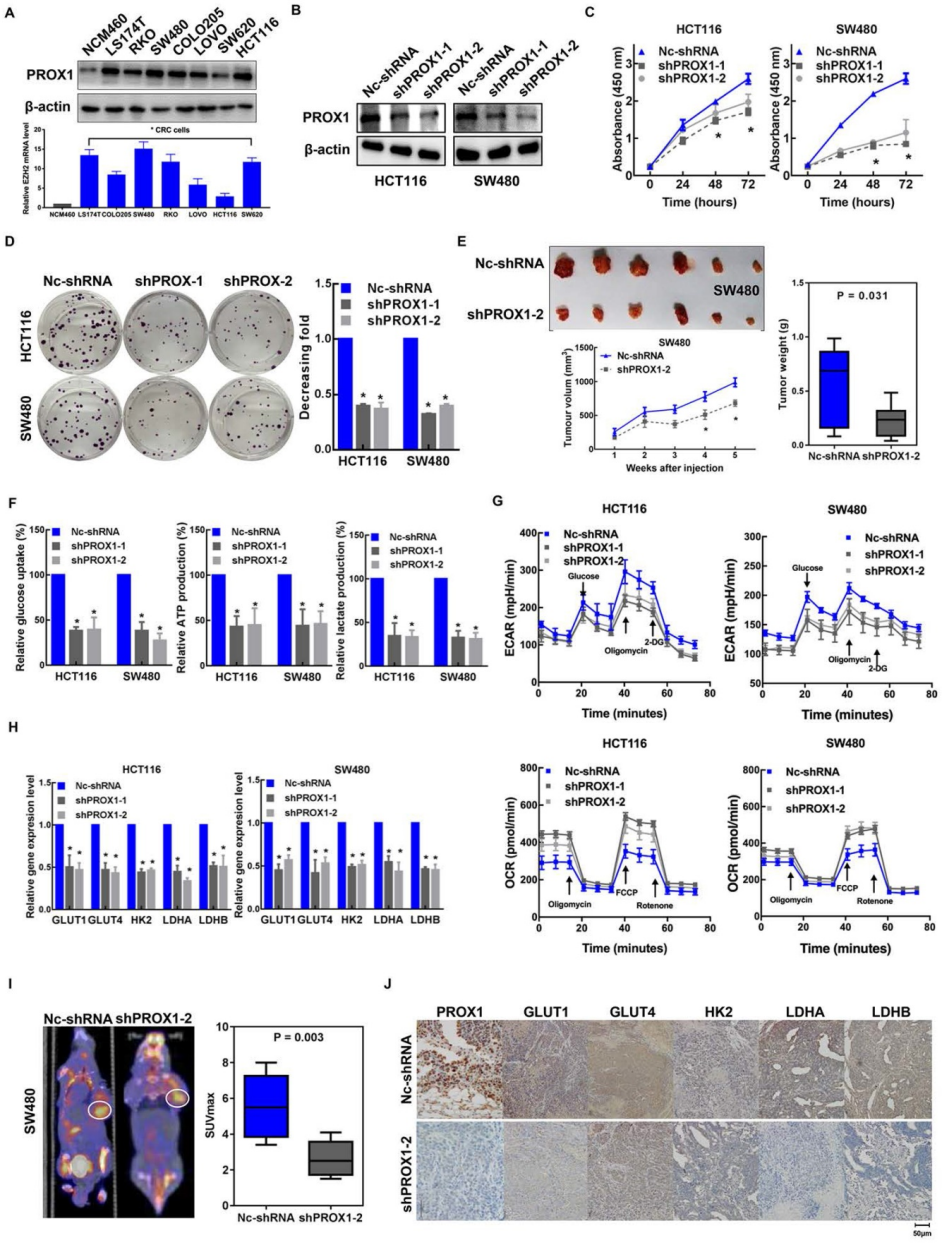
** PROX1 promotes CRC cell proliferation and glycolysis *in vitro* and* in vivo.* A.** PROX1 protein and mRNA expression in seven CRC cell lines and one normal colorectal epithelial line were determined using western blotting. β-actin was used as an internal control. * P < 0.05; according to Student's t test. **B.** The western blotting results show the efficiencies of PROX1 knockdown in HCT116 and SW480 cells. β-actin was used as an internal control. **C.** The CCK-8 assays showed the effect of Nc-shRNA and PROX1 knockdown on cell proliferation in HCT116 and SW480 cells. *: P < 0.05; according to Student's t test. **D.** Representative images (left panel) and quantitative analysis (right panel) of the colony formation assay results showed that knockdown of PROX1 suppressed cell proliferation in HCT116 and SW480 cells. *: P < 0.05; according to Student's t test. **E.** PROX1-knockdown or Nc-shRNA-transfected SW480 cells were injected into nude mice (n = 6) subcutaneously. Representative images of tumors are shown (upper). The nude mouse xenograft model showed that knockdown of PROX1 decreased tumor growth (lower left) and reduced tumor weights (right) compared with the Nc-shRNA groups. **F.** Glucose uptake, lactate production, and ATP levels in PROX1-knockdown and control HCT116 and SW480 cells were determined as described in the Materials and Methods. * P < 0.05; according to Student's t test. **G.** ECAR (an indicator of glycolysis) and OCR (reflecting mitochondrial respiration) were reduced in PROX1-knockdown HCT116 and SW480 cells. **H.** RT-qPCR analysis of the indicated rate-limiting enzymes in PROX1-knockdown and control HCT116 and SW480 cells. * P < 0.05; according to Student's t test. **I.** Representative photographs of 18F-FDG PET/CT scans of *xenograft* mice and analysis of the SUVmax in shPROX1 and Nc-shRNA groups. **J.** Representative images of the IHC analysis showed the expression of GLUT1, GLUT4, HK2, LDHA and LDHB in PROX1-knockdown and negative control SW480 cells transfected *xenograft* tumor tissue samples (200×).

Given the clinical association of PROX1 expression with glucose metabolism and tumor progression, we aimed to determine the specific effects of PROX1 on cell proliferation and glucose metabolism in CRC. To this end, we first measured the baseline PROX1 protein and mRNA expression levels in a panel of cell lines and found higher expression of PROX1 in CRC cells than in cells of the normal colonic epithelial cell line NCM460 (**Figure 2A**). The expression level of PROX1 was higher in HCT116 and SW480 cells than in other colon cancer cells, and these two cell lines were selected for further PROX1 knockdown experiments. We utilized two shRNAs to knock down PROX1 expression in HCT116 and SW480 CRC cells and confirmed PROX1 knockdown by western blotting (**Figure 2B**) and RT-qPCR (**Supplementary Figure 1A**). Consistent with the reported role of PROX1 in promoting cell proliferation, we found that PROX1-knockdown HCT116 and SW480 cells exhibited significantly slower *in vitro* proliferation and less colony formation than control cells (**Figure 2C-D**). Moreover, when transplanted into nude mice, *xenograft* tumors generated by PROX1-knockdown SW480 cells grew more slowly than those generated by control cells (**Figure 2E**). All these findings indicate that PROX1 may play a critical oncogenic role in CRC through its involvement in cell growth.

Given the observed role of PROX1 in modulating tumor growth and that glycolysis is the primary feature of metabolic reprogramming in cancer and is a metabolic signature for highly proliferative cancer, we speculated that PROX1 may promote cell proliferation by affecting glycolysis in colon cancer cells. As hypothesized, RNA sequencing and gene enrichment analysis of differentially expressed genes between PROX1-knockdown and control SW480 cells showed that the downregulated gene set was significantly enriched in genes involved in glycolysis (**Supplementary Figure 1B**). Next, we validated the RNA sequencing results in functional analyses of glucose metabolism using a Seahorse metabolic analyzer. The intracellular glucose uptake, lactate and ATP production, extracellular acidification rate (ECAR; an indicator of glycolysis) and oxygen consumption rate (OCR; reflects mitochondrial respiration) confirmed that knockdown of PROX1 suppressed glycolysis while promoting oxidative phosphorylation *in vitro* (**Figure 2F-G**). We also detected the influence of PROX1 on a list of rate-limiting glycolytic enzymes (GLUT1, GLUT4, HK2, LDHA and LDHB) by RT-qPCR, which showed that PROX1 knockdown markedly reduced the gene expression levels of these genes in HCT116 and SW480 cells (**Figure 2H**). To confirm these results under *in vivo* conditions, we harvested SW480 cell line-derived *xenograft* tumors and analyzed metabolic parameters. PET/CT analysis showed that PROX1 knockdown significantly suppressed glucose uptake by xenografted colon cancer cells and resulted in a decreased SUVmax (**Figure 2I**). We also performed IHC analysis of SW480 xenograft tumor tissues and confirmed that the protein levels of the glycolytic enzymes GLUT1, GLUT4, HK2, LDHA and LDHB were lower in *xenografts* from PROX1 knockdown cells than in those from control cells (**Figure 2J**). Taken together, these results suggest that PROX1 is a positive regulator of glycolysis in CRC.

### PROX1 inhibits the transcription of *SIRT3* in CRC

**Figure 3:**
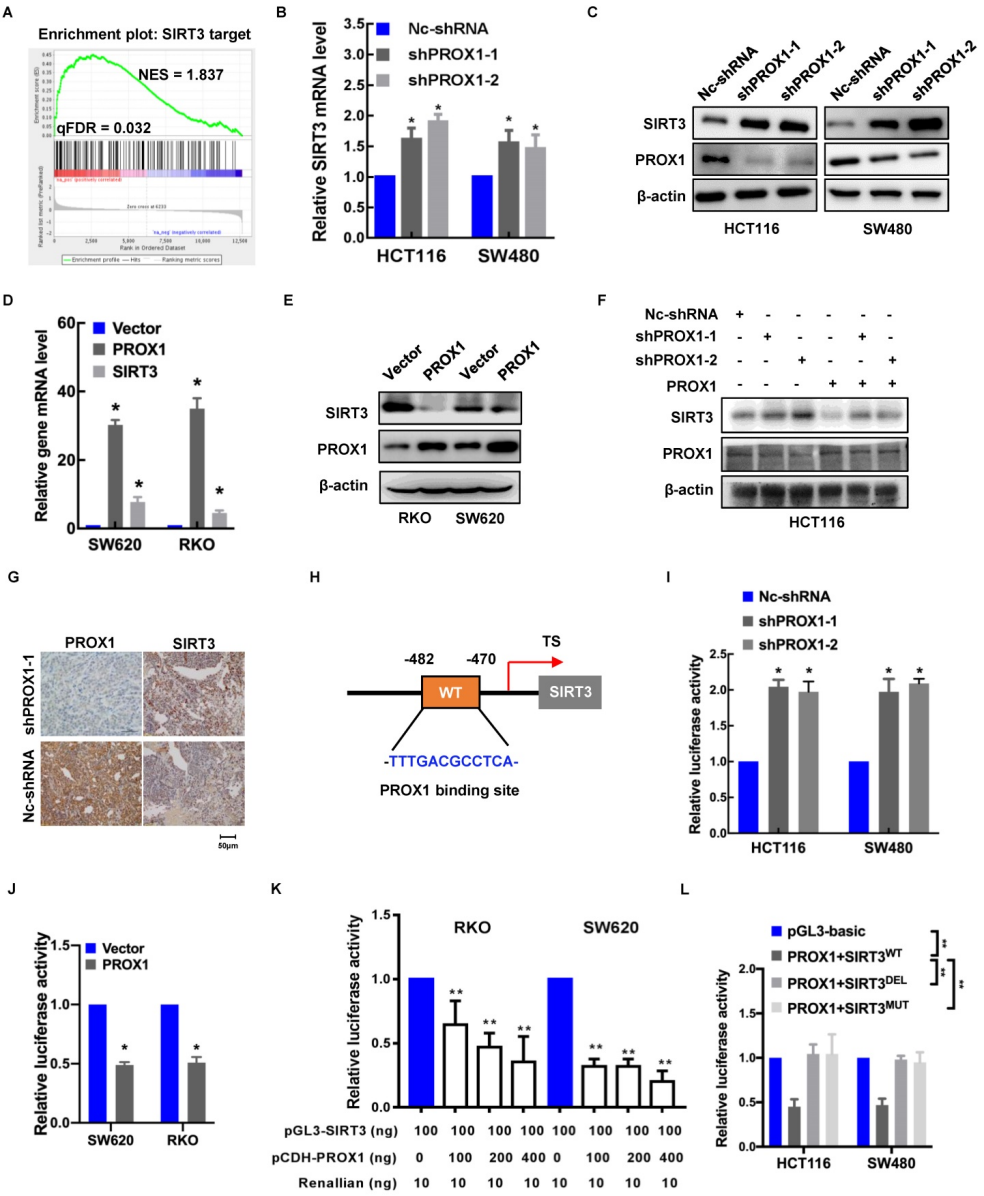
** PRXO1 epigenetically silences *SIRT3* expression. A.** GSEA of the differentially expressed genes in PROX1-knockdown and control SW480 cells showed high enrichment of *SIRT3* targets from RNA-sequencing data. **B.** RT-qPCR showed that the expression level of *SIRT3* was enhanced in PROX1-knockdown HCT116 and SW480 cells compared with negative control cells. *: P < 0.05; according to Student's t test. β-actin was used as an internal control. **C.** Western blotting showed that the expression level of *SIRT3* was enhanced in PROX1-knockdown HCT116 and SW480 cells compared with negative control cells. β-actin was used as an internal control. **D.** RT-qPCR showed that the expression level of *SIRT3* was reduced in PROX1-overexpressing RKO and SW620 cells compared with negative control cells. *: P < 0.05; according to Student's t test. β-actin was used as an internal control. **E.** Western blotting showed that the expression level of *SIRT3* was reduced in PROX1-overexpressing RKO and SW620 cells compared with negative control cells. β-actin was used as an internal control. **F.** Western blotting showed that the expression of SIRT3 protein in CRC cells with indicated treatment. β-actin was used as an internal control. **G.** Representative images of the IHC analysis showed the expression level of *SIRT3* in PROX1-knockdown and negative control SW480 cells transfected *xenograft* tumor tissue samples (200×). **H.** A schematic diagram showing the predicted PROX1-binding region in the human *SIRT3* upstream promoter. **I.** Dual-reporter luciferase assays showed that knockdown of PROX1 in HCT116 and SW480 cells stimulated *SIRT3* promoter reporter activity. *: P <0.05; according to Student's t test. **J.** Dual-reporter luciferase assays showed that overexpression of PROX1 in RKO and SW620 cells inhibited the activity of the *SIRT3* promoter reporter. *: P <0.05; according to Student's t test. **K.** The RT-qPCR results showed the efficiencies of PROX1 overexpression in RKO and SW620 cells. *: P<0.05; according to Student's t test. β-actin was used as an internal control. **L.** Dual-reporter luciferase assays showed the change in the promoter activity of *SIRT3* in RKO and SW620 cells with the indicated treatments. All white bars were compared to their left blue bars; **: P<0.01; according to Student's t test. **M.** Dual-reporter luciferase assays showed that compared to the wild-type (WT), PROX1 failed to stimulate the activity of the *SIRT3* promoter with deletion (DEL) of the predicted binding site. **: P<0.01; according to Student's t test.

To elucidate the mechanism underlying the effects of PROX1 on glucose regulation, we looked more closely at the genes involved in glucose metabolism that were potentially regulated by PROX1. Among the differentially expressed genes in PROX1-knockdown and control SW480 cells, *SIRT3* targets (including *SIRT3*) were enriched, indicating that *SIRT3* is a potential downstream regulator of PROX1 (**Figure 3A**). The mRNA level of SIRT3 in seven CRC cell lines also showed that *SIRT3* mRNA expression was to some extent negative correlated with *PROX1* mRNA expression (**Supplementary Figure 1C**). To confirm the screening results, we examined the effect of PROX1 on SIRT3 expression in colorectal cells. As shown in **Figure 3B-F**, PROX1 knockdown increased *SIRT3* expression at both the mRNA and protein levels, while PROX1 overexpression decreased *SIRT3* expression at both the mRNA and protein levels. In addition, SIRT3 protein expression was higher in *xenograft* tumors derived from PROX1-knockdown SW480 cells than in those from control SW480 cells (**Figure 3G**). These results suggest that PROX1 inhibits the transcription of *SIRT3* in CRC.

Given that PROX1 is a transcription factor, to investigate the regulatory mechanism underlying the correlation between PROX1 and *SIRT3* expression, we searched for possible PROX1 binding sites in the *SIRT3* promoter. Using the online software program JASPAR (http://jaspar.genereg.net/), we identified one putative PROX1 binding site in the region 482~470 bp upstream of the transcription start site in the *SIRT3* gene (**Figure 3H**). To assess whether PROX1 regulates *SIRT3* expression by binding to this site, we constructed a pGL3-*SIRT3*-promoter plasmid harboring a 1500-bp fragment (nucleotides -170~-1670) encompassing the predicted PROX1 binding site. Luciferase reporter assays indicated that *SIRT3* promoter activity was higher in PROX1-knockdown HCT116 and SW480 cells and lower in PROX1-overexpressing RKO and SW620 cells than in the corresponding controls (**Figure 3I-J**). In RKO and SW620 cells, which express little endogenous PROX1, luciferase reporter assays indicated that *SIRT3* promoter activity was lower in RKO and SW620 cells overexpressing PROX1 than in the corresponding controls (**Figure 3K**). Moreover, deletion and mutation of the predicted PROX1 binding site from the reporter plasmid eliminated the inhibitory effect of PROX1 on luciferase activity (**Figure 3L**). Collectively, these findings suggest that PROX1 binds to a putative site in the *SIRT3* promoter to suppress *SIRT3* transcription.

### PROX1 interacts with EZH2 to epigenetically silence *SIRT3* expression

**Figure 4:**
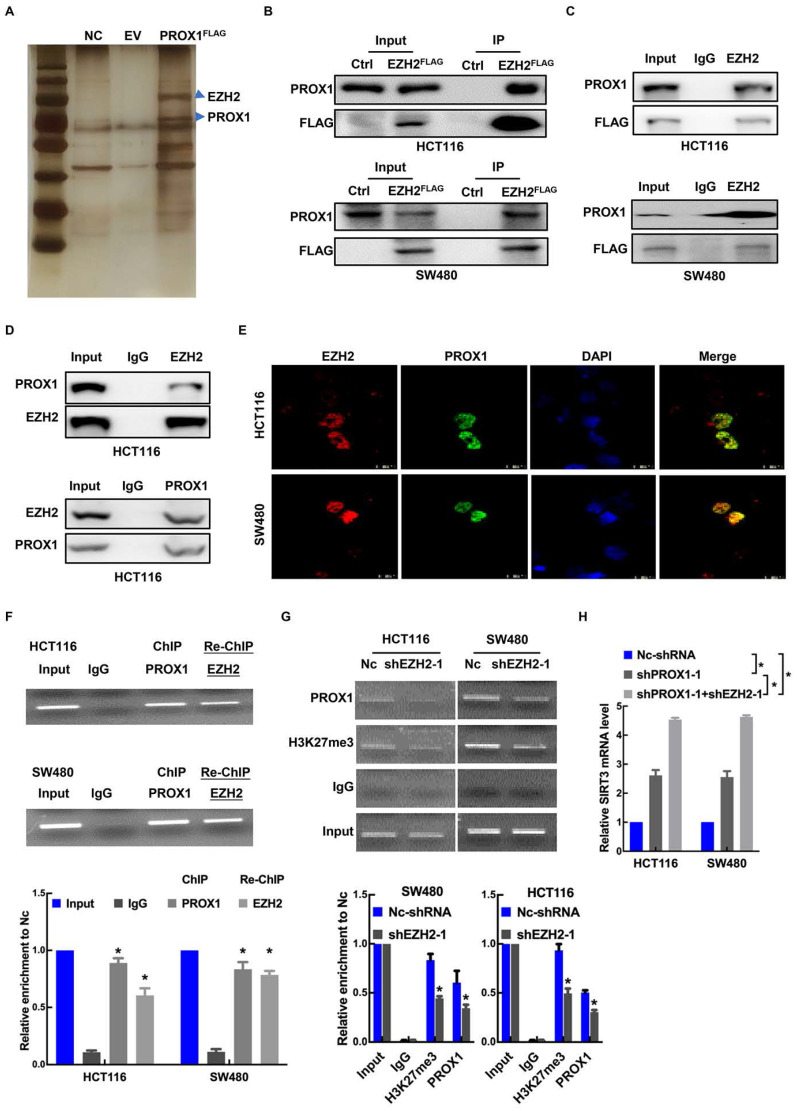
** PRXO1 interacts with EZH2 to epigenetically silence *SIRT3* expression. A.** Identification of PROX1‐associated factors using IP/MS. HEK293T cells were transfected with pFlag‐PROX1(PROX1^FLAG^) or empty vector (EV). **B.** The interaction between PROX1 and EZH2 was detected via exogenous co-IP assays. **C.** The interaction between PROX1 and EZH2 was detected via semiendogenous co-IP assays. **D.** The interaction between PROX1 and EZH2 was detected via endogenous co-IP assays. **E.** Representative immunofluorescence staining images showing the distribution and expression of PROX1 and EZH2 proteins in HCT116 and SW480 cells. **F.** Cells were harvested and formaldehyde fixed. After sonication, the chromatin was subjected to a ChIP assay using PROX1 antibodies. The eluted DNA was processed for re-ChIP with anti-EZH2 antibody or nonspecific IgG control. Input and coimmunoprecipitation DNA were analyzed by qPCR for the *SIRT3* promoter. *SIRT3* promoter segments were quantified using qRT-PCR against 5% input. The mean ± SD from three independent experiments is presented. **G.** ChIP assays showed that EZH2 knockdown suppressed the binding of PROX1 to the *SIRT3* promoter. IgG served as a negative control. *SIRT3* promoter segments were quantified using qRT-PCR against 5% input. The mean ± SD from three independent experiments is presented. **H.** RT-qPCR showed the expression of *SIRT3* in HCT116 and SW480 cells with different treatments. *: P <0.05; according to Student's t test.

Enhancer of zeste homolog 2 (EZH2), a histone-lysine N-methyltransferase enzyme, has been identified as an epigenetic corepressor 23, 25, 26. Previous studies have shown that PROX1 acts as a DNA-binding factor by interacting with other transcriptional coregulators 27. To explore the mechanism underlying PROX1's promotion of cell proliferation and glycolysis in CRC, GST pulldown was conducted to identify key factors associated with PROX1. FlaG-PROX1 produced in HEK293T cells was immunoprecipitated by antI-Flag mAb, and coprecipitated proteins were visualized by silver staining after electrophoresis and identified by MS. One coprecipitated factor was EZH2 (**Figure 4A**). We then performed exogenous and endogenous co-IP assays, which showed binding between PROX1 and EZH2 under all IP conditions (**Figure 4B-D**). As confirmation of the IP results, immunofluorescence assays revealed the cellular colocalization of PROX1 and EZH2 (**Figure 4E**). Thus, we proposed that PROX1 recruits EZH2 to the *SIRT3* promoter region to epigenetically silence *SIRT3* expression. To assess this, we performed sequential chromatin immunoprecipitation (ChIP/re-ChIP) assays in HCT116 and SW480 cells to examine PROX1 and EZH2 occupation at the *SIRT3* promoter region. As shown in **Figure 4F**, the ChIP results showed that PROX1 bound to the *SIRT3* promoter, and re-ChIP revealed that EZH2 bound to the PROX1-bound *SIRT3* promoter, suggesting that PROX1 recruits EZH2 to the *SIRT3* promoter region and modulates *SIRT3* expression. ChIP analysis of EZH2-knockdown and control cells showed that EZH2 knockdown decreased PROX1 levels at the *SIRT3* promoter region (**Figure 4G**). To investigate the effect of EZH2 on *SIRT3*, we knocked down EZH2 and assessed the effect of EZH2 on *SIRT3* expression. EZH2 mRNA and protein expression was successfully knocked down in HCT116 and SW480 cells (**Supplementary Figure 2A-B**), which increased *SIRT3* expression at the mRNA and protein levels (**Supplementary Figure 2A-B**). In addition, compared to the knockdown of PROX1 alone, simultaneous knockdown of PROX1 and EZH2 increased *SIRT3* mRNA levels (**Figure 4H**). Taken together, these results suggest that PROX1 can recruit EZH2 to the *SIRT3* promoter region in colon cancer cells, where EZH2 represses *SIRT3* transcription.

### EZH2 increased cancer cell proliferation and glycolysis in CRC

EZH2 is known to promote CRC carcinogenesis and progression 28, but its effect on glucose metabolism in CRC is unknown. Thus, we first analyzed the effects of EZH2 knockdown on HCT116 and SW480 cell proliferation and glucose metabolism. As anticipated, EZH2 knockdown decreased the proliferation rate and colony formation (**Supplementary Figure 2C-D**); decreased intracellular glucose uptake and lactate and ATP production (**Supplementary Figure 2E**); and reduced the ECAR but promoted the OCR (**Supplementary Figure 2F**). In addition, EZH2 knockdown markedly reduced the gene expression levels of GLUT1, GLUT4, HK2, LDHA and LDHB in HCT116 and SW480 cells (**Supplementary Figure 2G**). In the cohort of 217 CRC samples, 61.6% (n = 134) exhibited high EZH2 immunostaining, and 38.4% (n = 83) exhibited low EZH2 immunostaining (**Supplementary Figure 2H**). Analysis of the correlation of EZH2 levels with clinicopathological data for patients with CRC showed that high EZH2 expression was positively associated with tumor depth of invasion (P = 0.000), vascular invasion (P = 0.001), lymphatic metastasis (P = 0.000), TNM stage (P = 0.000) and Ki67 immunostaining (P = 0.000; **Table 1**). Moreover, patients with high EZH2 expression had significantly worse OS (P < 0.001) and DFS (P < 0.001) than patients with low EZH2 expression (**Supplementary Figure 2I**). By detecting EZH2 expression in the cohort of 71 patients who underwent PET/CT, we found that EZH2 expression was positively correlated with the SUVmax (**Supplementary Figure 2J**). These results suggest that EZH2 positively regulates glycolysis in CRC, with consequences on patient outcomes.

### *SIRT3* mediates the regulatory effects of PROX1 on cell proliferation and glycolysis in CRC

The previous results suggested that PROX1 might regulate glucose metabolism in CRC via *SIRT3* suppression. Therefore, to elucidate the role of *SIRT3* in CRC, this protein was overexpressed in HCT116 and SW480 cells via the transfection of *SIRT3* cDNA plasmid or control vector (**Supplementary Figure 3A-B**). *SIRT3* overexpression inhibited cell proliferation and colony formation (**Supplementary Figure 3C-D**) as well as glucose uptake and lactate and ATP production (**Supplementary Figure 3E**). As expected, the ECAR decreased significantly after *SIRT3* overexpression, while the OCR was significantly increased in *SIRT3*-overexpressing cells compared with control cells *in vitro* (**Supplementary Figure 3F**). In addition, SIRT3 overexpression markedly reduced the gene expression levels of GLUT1, GLUT4, HK2, LDHA and LDHB in HCT116 and SW480 cells (**Supplementary Figure 3G**). In the 217 human CRC tumor samples, *SIRT3* immunostaining was high in 51.6% (n = 112) and low in 48.4% (n = 105; **Supplementary Figure 3H**). Analysis of the correlation of *SIRT3* levels with clinicopathological data for patients with CRC showed that low *SIRT3* expression was positively associated with tumor depth of invasion (P = 0.012), lymphatic metastasis (P = 0.000) and TNM stage (P = 0.000; **Table 1**). Patients with high *SIRT3* expression had significantly better OS (P = 0.007) and DFS (P < 0.001) than patients with low *SIRT3* expression (**Supplementary Figure 3I**). Then, we analyzed the correlation between *SIRT3* immunostaining and the SUVmax in 71 patients who underwent PET/CT, and *SIRT3* immunostaining was inversely correlated with the SUVmax of patients with CRC (**Supplementary Figure 3J**). These results suggest that *SIRT3* negatively regulates glycolysis in CRC.

**Figure 5:**
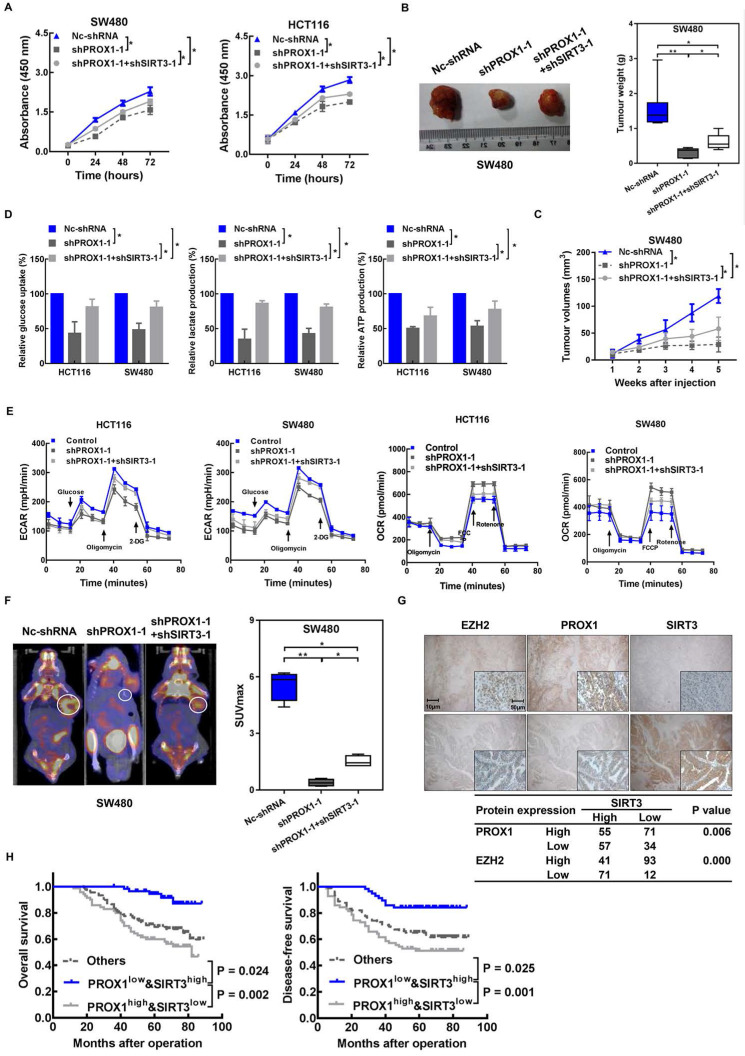
** Knockdown of *SIRT3* partly rescued PROX1 knockdown and induced a decrease in cell proliferation and glycolysis in CRC. A.** CCK-8 assay results showing changes in the cell proliferation rate after the knockdown of PROX1 with or without SIRT3 knockdown. * P < 0.05; according to Student's t test. **B.** PROX1-knockdown SW480 cells with or without SIRT3 knockdown or Nc-shRNA-transfected SW480 cells were injected into nude mice (n = 6) subcutaneously. Representative images of tumors are shown (left). The nude mouse *xenograft* model showed that *SIRT3* knockdown rescued the PROX1 knockdown-induced decrease in tumor weight (right). **C.** The nude mouse *xenograft* model showed that *SIRT3* knockdown rescued the PROX1 knockdown-induced decrease in tumor growth. **D.** Glucose uptake and lactate and ATP production were assessed after the knockdown of PROX1 with or without *SIRT3* knockdown. * P < 0.05; according to Student's t test. **E.** ECAR (an indicator of glycolysis) and OCR (reflecting mitochondrial respiration) in HCT116 and SW480 cells in Nc-shRNA and PROX1-knockdown SW480 cells with or without SIRT3 knockdown. **F.** Representative photographs of 18F-FDG PET/CT scans of *xenograft* mice and analysis of the SUVmax in Nc-shRNA and PROX1-knockdown SW480 cells with or without SIRT3 knockdown. **G.** Representative images of the IHC analysis showed the expression levels of *SIRT3*, PROX1 and EZH2 in tumor tissues from patients with CRC. The table shows that *SIRT3* expression was negatively correlated with PROX1 and EZH2 expression (χ^2^ test). **H.** Kaplan-Meier analysis of the correlation of combined PROX1 and *SIRT3* expression with OS and DFS. Log-rank tests were used to determine statistical significance.

Then, we investigated whether the effects of PROX1 on cell proliferation and glucose metabolism in CRC cells were mediated by *SIRT3*. The results showed that *SIRT3* knockdown (**Supplementary Figure 4A**) partially restored the growth (**Figure 5A-C**), capacity for glycolysis (**Figure 5D-F**) and mRNA level of glycolytic enzymes (**Supplementary Figure 4B**) of PROX1-knockdown HCT116 and SW480 cells *in vitro* and *in vivo*. Taken together, these results indicate that PROX1 promotes CRC cell proliferation and glucose metabolism remodeling in part via *SIRT3*.

Again, to provide preliminary indications of the clinical relevance of our identified mechanism of metabolic regulation in cancer involving PROX1, *SIRT3*, and EZH2, we examined the correlations between these protein expression levels in CRC patient tissues and clinical parameters and prognosis. Negative correlations were detected between *SIRT3* and both PROX1 (P = 0.006) and EZH2 (P = 0.000) by immunostaining (**Figure 5G**). Next, we assessed the prognostic potential of PROX1 and *SIRT3* expression in CRC and found that patients with low PROX1 and high *SIRT3* expression had much better OS (P = 0.002) and DFS (P = 0.001) than patients with other combinations of PROX1 and *SIRT3* expression (**Figure 5H**). Our results suggest that the combination of PROX1 and SIRT3 expression may be utilized as a powerful prognostication factor in CRC.

## Discussion

The transcription factor PROX1 has been implicated in CRC progression 29 and in cancer cell metabolism 17. Collectively, these previous discoveries suggested the role of PROX1 in metabolic reprogramming in cancer, but this hypothesis has not been conclusively demonstrated, and sufficient data are lacking on the specific role of PROX1 in CRC cell glucose metabolism. In the present study, we aimed to uncover the relationship between the expression profile and role of PROX1 and CRC cell glucose metabolism and to elucidate the underlying molecular mechanism. Here, clinical data, *in vitro* cell lines and *in vivo xenograft* mouse models were utilized in an in-depth exploration of the contribution of PROX1 to cancer cell metabolism and the underlying mechanism. We found that PROX1 regulates glycolysis and mitochondrial respiration, which are fundamental for sustaining cancer cell proliferation. Thus, the findings of our present study provide further evidence of the oncogenic role of PROX1 in CRC.

Since PROX1 was first linked to cancer through its ability to regulate cell differentiation 30, evidence for the importance of aberrant PROX1 expression in human malignancy has accumulated. More recent studies have shown that PROX1 expression is absent or drastically reduced in biliary system carcinomas 31 but preferentially upregulated in colon cancer 32; moreover, PROX1 is a direct target of the beta-catenin/TCF signaling pathway, which is responsible for neoplastic transformation of the colonic epithelium 10. These previous findings indicate a specific oncogenic role of PROX1 dysregulation in CRC. PROX1 is a potent oncogenic transcription factor that plays important roles in development and cell differentiation 9, 33, 34. In addition to modulating differentiation, the mechanism underlying the influence of PROX1 on other aggressive traits, such as increasing the proliferation and epithelial-mesenchymal transition of malignant cells 16, 32, and the role of PROX1 in the regulation of cholesterol metabolism 35, are also well illustrated. However, the mechanistic basis of its regulatory effect on target genes related to glucose metabolism remains poorly understood.

Given the lack of knowledge of metabolic genes regulated by PROX1, we performed RNA-sequencing analysis and identified *SIRT3* as a target potentially regulated by PROX1. *SIRT3* is the main mitochondrial deacetylase and plays important roles in metabolic homeostasis in normal cells 36. In some types of cancer, *SIRT3* functions as an oncogene, whereas in other types, it acts as a tumor suppressor, inducing cancer cell death under stress conditions 37. By identifying and validating the PROX1 binding site in the *SIRT3* promoter, we confirmed that PROX1 is the transcription factor responsible for decreased *SIRT3* expression in colon cancer cells; moreover, knocking down PROX1 expression increased *SIRT3* expression and reversed the malignant properties of CRC. In addition, the results of our functional experiments indicated that *SIRT3* partially attenuates PROX1-induced glucose metabolism. These results validate the PROX1-*SIRT3* axis as a promising new target for novel therapeutics for CRC. Further studies should examine the effects of small molecules that target PROX1 on cancer cell metabolism and *SIRT3* expression. Shi and colleagues confirmed that SIRT3 was downregulated in primary CRC samples 38, which was consistent with the data presented by Mi et al. 39 and our data. Notably, these results from real-world tissue samples contradicted the results of another study, which showed that SIRT3 was highly upregulated in CRC cells compared to a normal rectal mucosa cell line 40. Nevertheless, cancer cell lines derived from rectal sites are not representative of the overall expression profile of SIRT3 in CRC. Moreover, several studies have revealed that by being involved in mitochondrial function and antioxidant responses in colon cancer 40-43, SIRT3 promotes cancer cell viability, mobility and proliferation and contributes to chemoresistance in CRC, suggesting that the SIRT3 gene harbors pro-tumorigenic properties and can behave as an oncogene. Intriguingly, SIRT3 has also demonstrated tumor suppressor roles in CRC 39, 44, 45. SIRT3 can present cytotoxic properties by disturbing mitochondrial activity 39, 45; SIRT3 can also inhibit energy reprogramming by interrupting the Warburg effect 46. In addition, cancer is a multifactorial, multistep, complicated disease; numerous genes and proteins regulate each other and form complex interactions that result in cancer development and progression. These findings illustrate that SIRT3 may exhibit potential opposing roles during the development and progression of tumors in different intracellular environments and functional scenarios.

**Figure 6:**
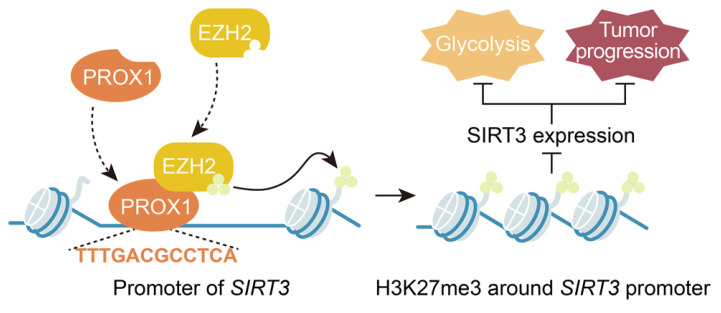
PROX1 recruits EZH2 to the SIRT3 promoter, thus inducing an increase in cell proliferation and glycolysis in CRC.

However, the mechanism by which PROX1 represses *SIRT3* expression remains unknown. PROX1 has been reported to suppress energy homeostasis in the liver by inhibiting the transcriptional regulatory activity of the ERRalpha/PGC-1alpha complex on metabolic genes 27. Thus, it was rational to hypothesize that PROX1 interacts with transcriptional coregulators or epigenetic modifiers to regulate *SIRT3* expression and thus modify cancer cell behavior. In this study, we provide the first evidence that PROX1 interacts with EZH2, a histone methyltransferase and one of the three core elements of polycomb repressive complex 2 (PRC2) 47, and recruits it to the *SIRT3* promoter, where EZH2 represses *SIRT3* transcription (**Figure 6**). EZH2 is a member of the polycomb group, whose constituents form two major core protein complexes, PRC1 and PRC2 48, that play important roles in differentiation, maintenance of cell identity, and proliferation 19, 23, 49-51 and are deregulated in a wide variety of cancers, in which they exert oncogenic or tumor-suppressive activity. The contribution of EZH2 to CRC has been thoroughly discussed, and this protein is a prognostic and therapeutic target in CRC 52-54; our study confirmed the prognostic value of EZH2 in the OS and DFS of CRC patients. To link to the regulatory effects of PROX1 on *SIRT3*, a protein known to regulate glucose metabolism, we showed that EZH2 promotes the remodeling of glucose metabolism in colon cancer cells, perhaps through its effects on *SIRT3*. These data imply that drugs targeting EZH2 may have downstream effects on cancer cell metabolism. Therefore, PROX1 engages EZH2 to downregulate SIRT3 to drive cellular glucose metabolism and cell proliferation.

In summary, our present study reveals a novel function of PROX1 in cancer cell metabolism, i.e., PROX1 recruits EZH2 to the *SIRT3* promoter and represses *SIRT3* transcription, which may represent the molecular mechanism underlying the observed biological consequences. Transcriptional complexes are complicated; our work has identified only one interacting molecule in the PROX1-related transcriptional complex, and further molecular identification work is needed to determine the other molecules involved in the PROX1-SIRT3 axis. Nevertheless, we believe that our findings highlight the ability of PROX1 to promote carcinogenesis by recruiting transcriptional coregulators to promote cancer cell proliferation and the expression of genes controlling glucose metabolism. Furthermore, PROX1, EZH2 and SIRT3 expression are potentially useful prognostic biomarkers in CRC, and targeting the PROX1-EZH2-*SIRT3* axis might present a novel therapeutic strategy for CRC. The results presented herein support the development of novel prognostic and therapeutic targets for CRC.

## Translational Relevance

PROX1 is an important transcription factor with an oncogene function in several types of tumors. However, the role of PROX1 in glucose metabolism remodeling in colorectal cancer (CRC) is unknown. In the present study, we found that PROX1 was markedly upregulated and positively correlated with tumor cell glycolysis in CRC. Furthermore, we described how PROX1 recruits EZH2 to the *SIRT3* promoter to silence promoter activity, which may reflect the molecular mechanism underlying its biological functions. Clinically, the expression level of PROX1 combined with that of *SIRT3* may be a useful prognostic biomarker in CRC, and targeting the PROX1-EZH2 complex axis might be a useful therapeutic strategy for CRC treatment.

## Supplementary Material

Supplementary methods and figures.

## Figures and Tables

**Table 1:** Relationship between protein expression and clinicopathological parameters in patients with CRC

Variable	Number (n = 217)	Patients with high PROX1 expression No. (%)	P value	Patients with low SIRT3 expression No. (%)	P value	Patients with high EZH2 expression No. (%)	P value
**Age (years)**			0.107		0.083		0.817
<60	70	35 (50.0%)		40 (57.1%)		44 (62.9%)	
≥60	147	91 (61.9%)		65 (44.2%)		90 (61.2%)	
**Sex**			0.889		0.785		0.535
Male	126	74 (58.7%)		62 (49.2%)		80 (63.5%)	
Female	91	52 (57.1%)		43 (47.3%)		54 (59.3%)	
**Location**			0.697		0.863		0.652
Ascending	86	46 (53.5%)		43 (50.0%)		55 (64.0%)	
Transverse	22	13 (59.1%)		12 (54.5%)		14 (63.6%)	
Descending	23	15 (65.2%)		11 (47.8%)		16 (69.6%)	
Sigmoid	86	52 (60.5%)		39 (45.3%)		49 (57.0%)	
**Histologic grade**			0.818		0.819		0.626
Well and moderately	196	113 (57.7%)		94 (48.0%)		120 (61.2%)	
Poorly and undifferentiated	21	13 (61.9%)		11 (52.4%)		14 (66.7%)	
**Tumor depth of invasion**			0.031*		0.012*		0.000*
T1, T2	38	16 (42.1%)		11 (28.9%)		11 (28.9%)	
T3, T4	179	110 (61.5%)		94 (52.5%)		123 (68.7%)	
**Vascular invasion**			0.282		0.050		0.001*
Absent	202	115 (56.9%)		94 (46.5%)		119 (58.9%)	
Present	15	11 (73.3%)		11 (73.3%)		15 (100%)	
**Lymphatic metastasis**			0.001*		0.000*		0.000*
Absent	125	61 (48.8%)		47 (37.6%)		63 (50.4%)	
Present	92	65 (70.7%)		58 (63.0%)		71 (77.2%)	
TNM stage			0.002*		0.000*		0.000*
I and II	121	59 (48.8%)		43 (35.5%)		59 (48.8%)	
III and IV	96	67 (69.8%)		62 (64.6%)		75 (78.1%)	
Ki67 expression			0.049*		0.077		0.000*
Negative	64	28 (43.8%)		24 (37.5%)		26 (40.6%)	
Weak	43	28 (65.1%)		19 (44.2%)		27 (62.8%)	
Moderate	50	33 (66.0%)		26 (52.0%)		36 (72.0%)	
Strong	60	37 (61.7%)		36 (60.0%)		45 (61.8%)	

*P < 0.05.

**Table 2:** Univariate and multivariate analyses of clinicopathological factors for overall survival in CRC

Variable	Univariate analysis		Multivariate analysis	
	HR (95% CI)	P value	HR (95% CI)	P value
Age (<60/≥60)	1.052 (0.630-1.757)	0.847		
Sex (Male/Female)	1.189 (0.730-1.937)	0.487		
Location (ascending, transverse/descending, sigmoid	1.020 (0.855-1.216)	0.828		
Histologic grade (well, mod/poor, undifferentiated)	1.764 (1.314-2.368)	0.000*	1.447 (1.055-1.985)	0.022*
Tumor depth of invasion (T1, T2/T3, T4)	3.286 (1.321-8.175)	0.011*		
Vascular invasion (present/absent)	5.119 (2.777-9.437)	0.000*		
Lymphatic metastasis (present/absent)	5.356 (3.137-9.143)	0.000*	3.906 (2.210-6.904)	0.000*
TNM stage (III+IV/I+II)	5.413 (3.118-9.397)	0.000*		
Ki67 (moderate, strong/negative, weak)	1.595 (1.285-1.980)	0.000*		
PROX1 (high/low)	3.166 (1.759-5.697)	0.000*	2.275 (1.244-4.161)	0.008*

HR Hazard ratio, CI Confidence interval, * Significance level: P < 0.05.

**Table 3:** Univariate and multivariate analyses of clinicopathological factors for disease-free survival in CRC

Variable	Univariate analysis		Multivariate analysis	
	HR (95% CI)	P value	HR (95% CI)	P value
Age (<60/≥60)	1.032 (0.637-1.671)	0.899		
Sex (Male/Female)	1.225 (0.772-1.942)	0.389		
Location (ascending, transverse/descending, sigmoid)	1.028 (0.871-1.214)	0.828		
Histologic grade (well, mod/poor, undifferentiated)	1.649 (1.233-2.206)	0.001*	1.489 (1.109-2.000)	0.008*
Tumor depth of invasion (T1, T2/T3, T4)	3.638 (1.469-9.012)	0.005*		
Vascular invasion (present/absent)	4.731 (2.575-8.628)	0.000*		
Lymphatic metastasis (present/absent)	4.332 (2.659-7.024)	0.000*	3.985 (2.445-6.497)	0.000*
TNM stage (III+IV/I+II)	4.468 (2.716-7.348)	0.000*		
Ki67 (moderate, strong/negative, weak)	1.556 (1.270-1.906)	0.000*	2.789 (1.427-5.450)	0.003*
PROX1 (high/low)	2.249 (1.361-3.715)	0.002*		

HR Hazard ratio, CI Confidence interval, * Significance level: P < 0.05.

## Abbreviations

CRC: colorectal cancer
OS: overall survival
MSI-H: microsatellite instability-high
PKM2: pyruvate kinase M2
PROX1: prospero-related homeobox 1
SUVmax: maximum standardized uptake value
FFPE: formalin-fixed paraffin-embedded
FUSCC: Fudan University Shanghai Cancer Center
WHO: World Health Organization
CPTAC: Clinical Proteomic Tumor Analysis Consortium
TMA: tissue microarray
ChIP: chromatin immunoprecipitation
FDG: fluorodeoxyglucose
PET/CT: positron emission tomography/computed tomography
GSEA: gene set enrichment analysis
DFS: disease-specific survival
IHC: immunohistochemistry
IRS: immunoreactive score
ECAR: extracellular acidification rate
OCR: oxygen consumption rate
EZH2: enhancer of zeste homolog 2
H3K27me3: histone H3 at lysine 27
PRC2: polycomb repressive complex 2
