# Supplementary material for: PROX1-mediated epigenetic silencing of SIRT3 contributes to proliferation and glucose metabolism in colorectal cancer
Source: Int J Biol Sci. 2023 Jan 1;19(1):50–65. doi: 10.7150/ijbs.73530 (PMC9760442; doi:10.7150/ijbs.73530)
Supplement: Supplementary file 1 — Supplementary methods and figures. [file ijbsv19p0050s1.pdf]

Supplemental Figure 1

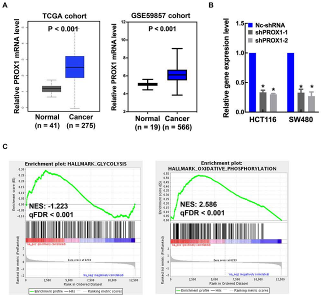

Supplementary Figure 2

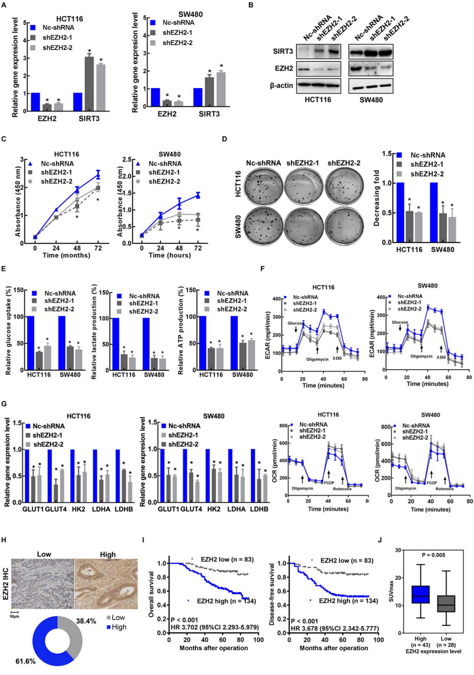

Supplementary Figure 3

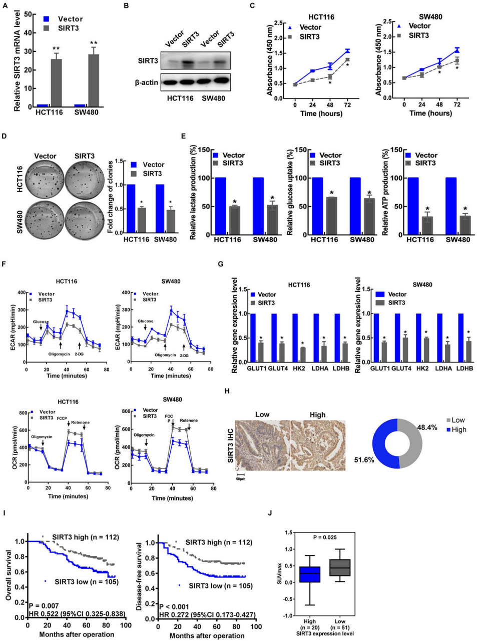

Supplemental Figure 4

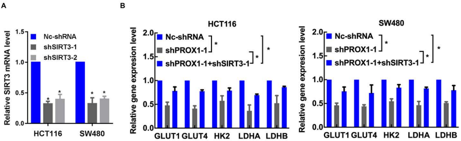

Supplementary materials

Cell lines and culture

Human colon cancer cell lines (LS174T, RKO, SW480, COLO205, LOVO, SW620 and HCT116) and normal colon epithelial cell line NCM460 were obtained from the Cell Bank of the Chinese Academy of Sciences. The identities of the cell lines were confirmed by DNA profiling (short tandem repeat (STR) sequencing). These cell lines were maintained in our laboratory and regularly subjected to routine cell line quality examinations (e.g., morphology and mycoplasma contamination) by HD Biosciences. All cells were maintained in 1640 medium (HyClone, Thermo Scientific, USA) supplemented with 10% fetal bovine serum (Gibco, Life Technologies, USA), 100 U/mL penicillin (Biowest, Nuaillé, France), and 100 U/mL streptomycin (Biowest, Nuaillé, France) and incubated at 37°C in a humidified atmosphere containing 5% CO_2.

Reagents and antibodies

Antibodies against PROX1 (11067-2-AP), H3K27me3 (39155), EZH2 (21800-1-AP), and SIRT3 (10099-1-AP) for chromatin immunoprecipitation (ChIP), IP and western blotting were obtained from Proteintech. Antibodies against β-actin and IgG were purchased from Cell Signaling Technology. Antibodies against GLUT1 (21829-1-AP), GLUT4 (21048-1-AP), HK2 (22029-1-AP), LDHA (19987-1-AP) and LDHB (14824-1-AP) for IHC were obtained from Proteintech. Polymerase II was purchased from Imgenex. All secondary antibodies were used at a 1:5000 dilution. ChIP assays were performed using a Pierce Agarose ChIP Kit (Thermo, #27177). Anti-flag (Sigma) secondary antibodies were conjugated to FITC or rhodamine (goat anti-rabbit IgG or goat anti-mouse IgG) (Jackson Laboratory) or peroxidase (goat anti-rabbit IgG or goat anti-mouse IgG) (Amersham Pharmacia Biotech). The primers and DNA oligos used in this study are listed in **Supplementary Table 1**.

Immunoprecipitation and mass spectrometry (IP/MS)

HEK293T cells transfected with pFlag-PROX1 were lysed in RIPA buffer (20 mM Tris pH 8.0, 137 mM NaCl, 1% NP40, 10% glycerol, 2 mM EDTA). The lysate was cleared by centrifugation at 12000 g before being loaded onto M2 anti-Flag mAb

agarose beads (Sigma, St. Louis, MO) preequilibrated in RIPA buffer. The beads were washed with RIPA buffer, and bound proteins were eluted using 3xFlag peptide (Sigma). Both eluants and postelution beads were boiled in loading buffer, resolved by SDS–PAGE and silver stained. Lysates from HEK293T cells transfected with empty vector were used as controls and processed in parallel. Bands specific to the pFlag-PROX1 transfection were excised and subjected to mass spectrometry analysis on ABI 4700 MALDI TOF/TOF.

Plasmid construction and viral infection

The PROX1 and EZH2 expression plasmids (pCDH-CMV-MCS-EF1-Puro-PROX1 and pCDH-CMV-MCS-EF1-Puro-EZH2) and the SIRT3 expression plasmid pcDNA3.1-SIRT3 were obtained from Transheep Technology (Shanghai, China). Short hairpin RNA (shRNA) constructs against PROX1 and EZH2 were generated using the pLKO.1-TRC cloning vector (Addgene, #10878). The primers and DNA oligos used in this study are listed in **Supplementary Table 1**. A shRNA sequence targeting firefly green fluorescent protein in the pLKO.1 vector was used as a control. Stably transfected CRC cells were established using puromycin selection after transfection with an expression vector or a control plasmid[19]. Lentivirus was produced by cotransfecting HEK-293T cells with pRSV-Rev, pMD2.G, pMDLg/pRRE and pCDH-puro expression vectors. Virus was harvested after 48 hours by filtering the virus-containing medium through a 0.45-μM Steriflip filter (Millipore). The full human cDNA sequences of PROX1 and EZH2 were purchased from Transheep Biosciences (Shanghai, China). The cDNA sequence of SIRT3 was subcloned into a pCDNA3.1 vector. The potential transcription factor-binding sites in the promoter regions of SIRT3 were predicted using GeneCards software (www.genecards.org) and the UCSC Genome Bioinformatics Site (http://genome.ucsc.edu/). The promoter sequence of SIRT3 (-1670~-170 bp) was cloned from genomic DNA prepared from SW620 cells using a Genomic DNA Extraction Kit (Tiangen Biotech Co. Ltd., Beijing). Then, the promoter sequences of SIRT3 were subcloned into the pGL3-basic vector to generate the recombinant plasmid pGL3-SIRT3-promoter.

Real-time reverse transcription quantitative polymerase chain reaction (RT–qPCR) and western blot analysis

Total RNA was extracted with TRIzol (Invitrogen) according to the manufacturer's instructions. Real-time qRT–PCR and western blot assays were performed as previously described[19].

Protein purification

Recombinant plasmids constructed from pGEX-4T3 and pET-28a that harbored the indicated GST-tagged (stat3 functional regions 1-6) and His-tagged (fbp1 exons 1-7) proteins, respectively, were separately transformed into BL21(DE3) chemically competent *E. coli* (Tiangen Biotech, China). Protein expression was induced by adding 1 mM isopropyl β-D-1-thiogalactopyranoside (IPTG) (Takara Biotechnology, Shiga, Japan) to Luria-Bertani (LB) medium and rocking at 16°C for 20 hours. Bacterial pellets were collected and resuspended in PBS buffer supplemented with 100 μg/mL protease inhibitor (Roche Diagnostics) and sonicated on ice for 15-second pulses at a high intensity (35 Hz) (BioTeK). Then, the samples were centrifuged, and the supernatant protein mixtures were collected for further purification.

GST-tagged and His-tagged proteins were further purified using GST or Ni-NTA Sefinose Resin kits (Sangon Biotech, China) following the manufacturer's protocol. Briefly, a mixture of the protein extract with binding buffer was added to columns filled with resin, which has a high affinity for GST-tagged or His-tagged recombinant proteins. Nonspecifically bound proteins were eliminated by washing the resin until the absorbance of the flowthrough fraction at 280 nm approached the baseline value. Then, recombinant proteins were eluted from the resin with elution buffer. Protein elution was monitored by measuring the absorbance of the fractions at 280 nm. Finally, the eluted proteins were analyzed by sodium dodecyl sulfate–polyacrylamide gel electrophoresis (SDS–PAGE).

Immunoprecipitation and coimmunoprecipitation

Cells were collected and lysed in radioimmunoprecipitation assay (RIPA) lysis buffer (Beyotime) containing a protease inhibitor cocktail (Roche Diagnostics).

Whole-cell lysates (2 mg) were precleared with 30 μL of protein G beads (Life Technologies), and 2 μg of isotype-matched IgG control or antibodies was then added and incubated for 2 hours on a rocking platform. Immunoprecipitants were collected by centrifugation and were then resolved by SDS–PAGE.

Cell proliferation and colony formation assay

The CCK-8 assay and colony formation assay were performed as previously described[19].

Glycolysis analysis

The cellular glycolysis rate was determined by a Glucose Uptake Colorimetric Assay Kit and Lactate Colorimetric Assay Kit (BioVision). RT–PCR was used to determine the expression levels of glycolytic enzymes in the cells[20].

Analysis of ATP production

The ENLITEN ATP Assay System (Promega; FF2000) was used according to the manufacturer's instructions. Cells were harvested by scraping and resuspended in PBS. The cell suspension was divided into unequal aliquots. Part of the cell suspension was mixed with 5% trichloroacetic acid (TCA). The remaining cells were used for the cell number calculation. Tris-acetate buffer (pH 7.75) was then added to neutralize the TCA and to dilute the TCA to a final concentration of 0.1%. The diluted sample (40 mL) was added to an equal volume of rL/L reagent (Promega; FF2000). Then, luminescence was measured. The ATP standard (Promega; FF2000) was serially diluted to generate a regression curve for calculating ATP concentrations in individual samples. The relative ATP concentration was determined and normalized to that of the control cells, which was designated as 1.

Oxygen consumption rate (OCR) and extracellular acidification rate (ECAR) measurements

Cellular mitochondrial function was measured using a Seahorse XF Cell Mito Stress Test Kit and a Bioscience XF96 Extracellular Flux Analyzer according to the manufacturer's instructions. The glycolytic capacity was determined using a Glycolysis Stress Test Kit (ab222946, Abcam) according to the manufacturer's

instructions. Briefly, 4 ×10^4 cells were seeded in 96-well plates and incubated overnight. After washing the cells with Seahorse buffer (DMEM with phenol red containing 25 mmol/L glucose, 2 mmol/L sodium pyruvate, and 2 mmol/L glutamine), 175 mL of Seahorse buffer plus 25 mL each of 1 mmol/L oligomycin, 1 mmol/L FCCP, and 1 mmol/L rotenone was automatically injected to measure the OCR. Then, 25 mL each of 10 mmol/L glucose, 1 mmol/L oligomycin, and 100 mmol/L 2-deoxyglucose was added to measure the ECAR. The OCR and ECAR values were calculated after normalization to the cell number and are plotted as the means ± SDs.

Animal studies

Animal experiments were approved by the Ethics Committee at Fudan University Shanghai Cancer Center (FUSCC) and performed as previously described[19,21]. Sections of the specimens (4 μm) were cut for IHC staining.

Immunofluorescence staining

Immunofluorescence staining was performed as previously described [20].

Supplementary Figure 1. Knockdown efficiencies and RNA-sequencing data

A. The RT–qPCR results show the efficiencies of *PROX1* knockdown in HCT116 and SW480 cells. *: P <0.05; according to Student's t test.

B. GSEA of the differentially expressed genes in PROX1-knockdown and control SW480 cells showed high enrichment of glycolysis and the oxidative phosphorylation pathway from RNA-sequencing data.

C. ROX1 and SIRT3 mRNA expression in seven CRC cell lines were determined using western blotting. β-actin was used as an internal control.

Supplementary Figure 2. The oncogene EZH2 is a positive regulator of glucose metabolism reprogramming in CRC

A. The RT–qPCR results show the efficiencies of EZH2 knockdown and its effect on SIRT3 expression in HCT116 and SW480 cells. *: P<0.05; according to Student's t test.

B. The western blotting results show the efficiency of EZH2 knockdown and its effect on SIRT3 expression in HCT116 and SW480 cells. β-actin was used as an internal control.

C. The CCK-8 assays showed the effect of EZH2 knockdown on cell proliferation in HCT116 and SW480 cells. *: P < 0.05; according to Student's t test.

D. Representative images (left panel) and quantitative analysis (right panel) of the colony formation assay results showed the effect of EZH2 knockdown on cell proliferation in HCT116 and SW480 cells. *: P < 0.05; according to Student's t test.

E. Glucose uptake, lactate production, and ATP levels in EZH2-knockdown and control HCT116 and SW480 cells were determined as described in the Materials and Methods. * P < 0.05; according to Student's t test.

F. ECAR and OCR in EZH2-knockdown and control HCT116 and SW480 cells.

G. RT–qPCR analysis of the indicated rate-limiting enzymes in EZH2-knockdown and control HCT116 and SW480 cells. * P < 0.05; according to Student's t test.

H. Representative images (upper panel) and distribution analysis (lower panel) of EZH2 immunohistochemical staining in CRC and normal colorectal samples

at various levels (n = 217; 200×).

I. Kaplan–Meier analysis of the correlation of EZH2 expression with OS and DFS. Log-rank tests were used to determine statistical significance.

J. Analysis of the SUVmax in CRC samples with low and high EZH2 IHC scores (n = 71).

Supplementary Figure 3. The tumor suppressor SIRT3 is a negative regulator of glucose metabolism reprogramming in CRC

A. The RT–qPCR results show the efficiencies of SIRT3 overexpression in HCT116 and SW480 cells. *: $P<0.05$; according to Student's t test.

B. The western blotting results show the efficiencies of SIRT3 overexpression in HCT116 and SW480 cells. β-actin was used as an internal control.

C. The CCK-8 assays showed the effect of the empty vector and overexpression of SIRT3 on cell proliferation in HCT116 and SW480 cells. *: $P < 0.05$; according to Student's t test.

D. Representative images (left panel) and quantitative analysis (right panel) of the colony formation assay results showed the effect of the empty vector and overexpression of SIRT3 on cell proliferation in HCT116 and SW480 cells. *: $P < 0.05$; according to Student's t test.

E. Glucose uptake, lactate production, and ATP levels in SIRT3-overexpressing and control HCT116 and SW480 cells were determined as described in the Materials and Methods. * $P < 0.05$; according to Student's t test.

F. ECAR and OCR in the presence of SIRT3 overexpression and control HCT116 and SW480 cells.

G. RT–qPCR analysis of the indicated rate-limiting enzymes in SIRT3-overexpressing and control HCT116 and SW480 cells. * $P < 0.05$; according to Student's t test.

H. Representative images (upper panel) and distribution analysis (lower panel) of SIRT3 immunohistochemical staining in CRC and normal colorectal samples at various levels (n = 217; 200×).

I. Kaplan–Meier analysis of the correlation of SIRT3 expression with OS and DFS.

Log-rank tests were used to determine statistical significance.

J. Analysis of the SUVmax in CRC samples with low and high SIRT3 IHC scores (n = 71).

Supplementary Figure 4. Knockdown efficiencies

A. The RT–qPCR results show the efficiencies of SIRT3 knockdown in HCT116 and SW480 cells. *: $P < 0.05$; according to Student's t test.

B. RT–qPCR analysis of the indicated rate-limiting enzymes in PROX1-shRNA and/or SIRT3-overexpressing and Nc-shRNA treated HCT116 and SW480 cells. * $P < 0.05$; according to Student's t test.
